# Expression of glutathione, glutathione peroxidase and glutathione S-transferase pi in canine mammary tumors

**DOI:** 10.1186/1746-6148-10-49

**Published:** 2014-02-24

**Authors:** Camila Leonel, Gabriela B Gelaleti, Bruna V Jardim, Marina G Moschetta, Vitor R Regiani, Juliana G Oliveira, Debora APC Zuccari

**Affiliations:** 1 Graduate Program in Genetics, Universidade Estadual Paulista – UNESP/IBILCE, São José do Rio Preto, SP, Brazil; 2 Graduate Program in Health Sciencies, Laboratory of Molecular Reserach in Cancer (LIMC), Departament of Molecular Biology, Faculdade de Medicina de São José do Rio Preto, FAMERP, São José do Rio Preto, SP, Brazil; 3 Graduate Program in Health Sciencies, Research Unit Genetics and Molecular Biology (UPGEM), Faculdade de Medicina de São José do Rio Preto, FAMERP, São José do Rio Preto, SP, Brazil; 4 Universidade do Sagrado Coração – USC, Bauru, SP, Brazil; 5 Departament of Molecular Biology, Faculdade de Medicina de São José do Rio Preto, FAMERP, São José do Rio Preto, SP, Brazil

**Keywords:** Mammary neoplasia, Glutathione, Immunohistochemistry, Real-time PCR, Oxidative stress

## Abstract

**Background:**

Glutathione (GSH) is one of the most important agents of the antioxidant defense system of the cell because, in conjunction with the enzymes glutathione peroxidase (GSH-Px) and glutathione S transferase pi (GSTpi), it plays a central role in the detoxification and biotransformation of chemotherapeutic drugs. This study evaluated the expression of GSH and the GSH-Px and GSTpi enzymes by immunohistochemistry in 30 canine mammary tumors, relating the clinicopathological parameters, clinical outcome and survival of the bitches. In an *in vitro* study, the expression of the genes glutamate cysteine ligase (*GCLC*) and glutathione synthetase (*GSS*) that synthesize GSH and *GSH-Px* gene were verified by qPCR and subjected to treatment with doxorubicin, to check the resistance of cancer cells to chemotherapy.

**Results:**

The immunohistochemical expression of GSH, GSH-Px and GSTpi was compared with the clinical and pathological characteristics and the clinical outcome in the bitches, including metastasis and death.

The results showed that high immunoexpression of GSH was correlated to the absence of tumor ulceration and was present in dogs without metastasis (*P* < 0.05). There was significant correlation of survival with the increase of GSH (*P* < 0.05). The expression of the GSH-Px and GSTpi enzymes showed no statistically significant correlation with the analyzed variables (p > 0.05). The analysis of the relative expression of genes responsible for the synthesis of GSH (GCLC and GSS) and GSH-Px by quantitative PCR was done with cultured cells of 10 tumor fragments from dogs with mammary tumors.

The culture cells showed a decrease in *GCLC* and *GSS* expression when compared with no treated cells (*P* < 0.05). High GSH immunoexpression was associated with better clinical outcomes.

**Conclusion:**

Therefore, high expression of the GSH seems to play an important role in the clinical outcome of patients with mammary tumors and suggest its use as prognostic marker. The *in vitro* doxorubicin treatment significantly reduces the expression of *GCLC* and *GSS* genes so we can consider them to be candidates for predictive markers of therapeutic response in mammary cancer.

## Background

Clinical and molecular characteristics of tumors may assist in the development of more efficient and less toxic therapeutic strategies. In this way, the expression of antioxidant proteins in tumor cells has been investigated as a predictor and prognostic factor in cytotoxic treatment in breast cancer [1].

When there is an excessive production of reactive oxidative species (ROS), the antioxidant defense system is triggered, which is of great importance in the physiopathology of diverse cancer types, including breast cancer [2,3]. The consequence of this process is the loss of cellular function and progression towards cell death. Moreover, oxidative stress leads to DNA damage and mutations in tumor suppressor genes, events that can be important in the initiation of carcinogenesis [4].

GSH is a tripeptide of glycine, cysteine and glutamic acid that is found in high concentration intracellularly, being the most abundant low molecular mass thiol [5]. Synthesis of GSH requires the consecutive action of two enzymes, first glutamate-cysteine ligase (GCLC) that conjugates glutamic acid and cysteine forming gama-glutamyl cysteine. This compound containing cysteine with a sulfhydryl group (SH) is responsible for the antioxidant activity of GSH. The second reaction is the binding of gama-glutamyl cysteine with glycine by the enzyme glutathione synthetase (GSS), giving rise to the tripeptide gama-glutamyl cysteine glycine-glutathione [6,7].

GSH-Px is an 80 kDa protein composed of four identical sub-units. Five isoforms of GSH-Px are well characterized in mammals and show tissue-specific distribution. Alteration of these enzyme levels are associated with diverse cancer types, including skin, kidney, intestine and breast cancer [8].

GSTs are a family of intracellular enzymes of detoxification phase II that catalyze diverse electrophilic compounds conjunction to GSH, blocking DNA mutations of the cells [9].

Admittedly, the classification of mammary neoplasms in female dogs is complex and detailed, but it cannot predict the progress of the illness and the survivor time of the animal with cancer nor the chemotherapeutic and/or hormonal treatment feedback. Nowadays, using the possibility of immunohistochemistry diagnosis of the tumors by biomarkers, prognosis can be more accurate and its predictable value can also direct genetic treatment. Besides, the biomarkers that apparently do not have independent prognostic value, together with the clinic-pathologic parameters, may assist in the evaluation of feedback to the patient’s clinical development.

Despite advances in identification of genes involved in tumor growth, progression and resistance to drugs, the way these genes interact with one another remains unclear. Therefore a better understanding of how molecular markers are associated with therapeutic response is indispensable in selecting the correct drugs for treatment. The capacity to establish primary cell cultures of tumors is an important requirement in cancer research, allowing the identification of important prognostic factors and therapeutic agents. Moreover, this is the best way of assessing the predictive value of adjuvant treatment that might also be given to cancer patients [10].

Primary cultures have been successfully used to get answers to drug dosages and to support new anti-cancer drug development [11]. The enzymatic system of glutathione/glutathione transferases (GSH/GSTs) is seen as one of the most important in cellular resistance to multiple drugs (MDR), involving the alteration of GSH level, the expression of genes that codify the enzymes responsible for its synthesis, and the genes that code for GSTs [5].

The aim of this study has been to assess the expression of GSH and the enzymes GSTpi and GSH-Px by immunohistochemistry in mammary tumors and to infer their prognostic value. An *in vitro* study of molecular analyses was made, verifying the resistance of neoplastic cells to the chemotherapeutic agent.

## Results

### Clinical data

Bitches from the test group were evaluated with respect to physical (age), pathological (Histological type, time course – interval between tumor diagnosis and surgical removal, number of nodules, clinical staging, ulceration and vascularization) and clinical (metastasis, censure) characteristics. The animals ranged from 6 to 16 years of age, the mean at diagnosis being 10 years. With respect to the breed, there were 12 (40%) mongrels, eight (27%) poodles, three (10%) cockers and three (10%) boxers, two (7%) pinchers, one (3%) basset and one (3%) dachshund. A total of 30 canine mammary lesions (6 benign and 24 malignant tumors) were histopathologically diagnosed, the benign tumors being composed of three mixed benign tumors, two sarcomatoid-like lesion and one papilloma while the malignant tumors included 14 carcinomas in mixed tumors, three papillary carcinomas, two carcinosarcomas, two tubulopapillary carcinomas, one comedocarcinoma, one solid carcinoma and one inflammatory carcinoma. There was a predominance of clinical staging I (37%). Among the clinicopathological characteristics, 21 (70%) of the tumors had multiple numbers of nodules, 18 (60%) tumors had the absence of ulceration and 15 (50%) had moderate vascularization. The metastasis and death index was 27% where all the cases of death occurred from pulmonary metastasis. All the data were described in Table 1.

**Table 1:** Correlation between the three antibody stains with the clinic and pathologic factors of the female dogs

**Clinical and pathologic factors**	**Number of bitches**	**GSH**	**GSTpi**	**GSH-Px**
**Age**				
≥ 10 years	16 (53.3%)	185.3 ± 6.532	202.8 ± 2.537	194.4 ± 3.838
< 10 years	14 (46.7%)	196.9 ± 6.170	206.5 ± 1.842	202.3 ± 2.936
P		> 0.05	> 0.05	> 0.05
**Time course**				
Until 6 months	13 (50%)	196.5 ± 21.82	204.6 ± 6.172	202.9 ± 13.81
Between 6 and 18 months	7 (27%)	182.3 ± 31.20	205.5 ± 9.397	192.5 ± 13.55
More than 18 months	6 (23%)	180.0 ± 24.07	202.5 ± 14.49	190.8 ± 14.18
P		> 0.05#	> 0.05#	> 0.05#
**Histological type**				
Malignant tumors	26 (87%)	189.9 ± 4.829	204.0 ± 1.757	198.3 ± 2.869
Benign	4 (13%)	196.3 ± 15.69	209.3 ± 3.860	203.5 ± 4.173
P		> 0.05	> 0.05	> 0.05
**Clinical staging**				
I	11 (37%)	202.3 ± 15.36	204.7 ± 5.746	199.4 ± 11.83
II	4 (13%)	190.0 ± 45.03	208.0 ± 4.243	198.3 ± 19.57
III	10 (33%)	183.3 ± 24.50	202.2 ±12.75	196.4 ± 13.09
IV	5 (17%)	180.6 ± 21.13	206.0 ± 8.689	198.4 ± 18.94
P		> 0.05#	> 0.05#	> 0.05#
**Number of nodules**				
Multiples	9 (30%)	177.9 ± 7.181	204.6 ± 2.844	200.8 ± 5.112
Single	21 (70%)	196.2 ± 5.314	204.5 ± 2.001	197.4 ± 3.044
P		> 0.05	> 0.05	> 0.05
**Tumor ulcerated**				
Ulceration	10 (34%)	180.8 ± 22.75	203.5 ± 13.53	205.0 ± 9.129
Necrosis	2 (7%)	162.0 ± 55.15	204.9 ± 5.557	189.5 ± 6.364
Absent	17 (59%)	199.4 ± 19.52	206.0 ± 8.485	195.2 ± 15.34
P		0.03* #	> 0.05#	> 0.05#
**Tumor vascularization**				
Abundant	15 (50%)	184.1 ± 7.164	205.7 ± 2.080	197.9 ± 3.349
Moderate	15 (50%)	197.3 ± 5.374	203.3 ± 2.499	198.2 ± 3.914
P		> 0.05	> 0.05	> 0.05
**Censure**				
Death	8 (26.7%)	165.5 ± 8.592	204.1 ± 1.869	203.0 ± 3.937
Living	22 (73,3%)	199.9 ± 3.921	205.9 ± 1.191	196.3 ± 3.099
P		0.0003*	> 0.05	> 0.05
**Metastasis**				
Yes	8 (26.7%)	165.5 ± 8.592	204.1 ± 1.869	203.0 ± 3.937
No	22 (73.3%)	199.9 ± 3.921	205.9 ± 1.191	196.3 ± 3.099
P		0.0003*	> 0.05	> 0.05

*Significant value to Student’s *t* test # ANOVA test.

### Immunohistochemistry study

**Figure 1:**
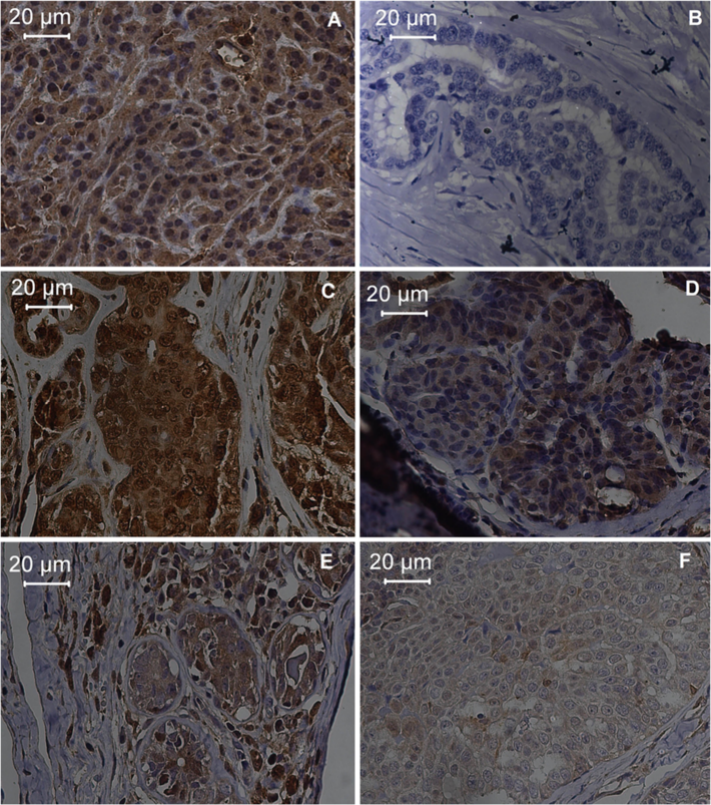
**Photomicrograph of the immunohistochemistry procedure demonstrating antibody staining in female dogs with mammary tumors.** AXIOSKOP2, 40X. **A**. GSH Immunostaining (medium intensity). **B**. GSH Immunostaining (negative). **C**. GSH-Px Immunostaining (high intensity). **D**. GSH-Px Immunostaining (low intensity). **E**. GSTpi Immunostaining (high intensity). **F**. GSTpi Immunostaining (medium intensity).

Immunostaining of GSH was evident in the cytoplasm of neoplastic cells, and GSTpi and GSH-Px were also focally seen in the nuclei. Both antibodies labeled the stroma (Figure 1).

There was no correlation between the expression of the GSH and clinical characteristics and pathology with the age, time course, histological type, clinical staging, number of nodules and tumor vascularization (*P* > 0.05; Table 1). However, the high expression of GSH had statistically significant correlation in female dogs with non-ulcerated tumors (*P* = 0.03; Table 1).

The expression of GSH, GSTpi and GSH-Px by immunohistochemistry was compared with the clinical development of the female dogs, including metastasis and death. The metastasis index was 26.7% and the same for the death index (Table 1). GSH expression increased in female dogs that survived to the end of follow-up (*P* = 0.0003; Table 1) and it also increased in those female dogs that did not present metastasis (*P* = 0.0003; Table 1). There was no significant statistical relation between the analyzed variables and the expression of the enzymes GSTpi and GSH-Px (*P* > 0.05; Table 1).

To generate the ROC curve, the values of the GSH, GSTpi and GSH-Px expression of the female dogs that died were compared to those that survived. The ROC curves indicate the performance and the limit values for GSH, GSTpi and GSH-Px expression in predicting the risk of mortality, and calculated the sensitivity/specificity, thereby establishing the best cut-off to discriminate high-death-risk dogs, which for GSH expression was: M.D.O. = 197 u.a. (sensitivity = 100% and specificity = 70%), GSTpi expression was: M.D.O. = 212 u.a. (sensitivity = 43% and specificity = 88%) and GSH-Px expression: M.D.O. 197 u.a. (sensitivity = 71% and specificity = 59%).

The dogs were followed-up from 1–18 months for overall survival, with a median of 540 days. The relationship between GSH, GSH-Px and GSTpi enzyme expression and survival of patients was evaluated by Kaplan–Meier analysis and log-rank test, using the cut-off value established by the ROC curve.

**Figure 2:**
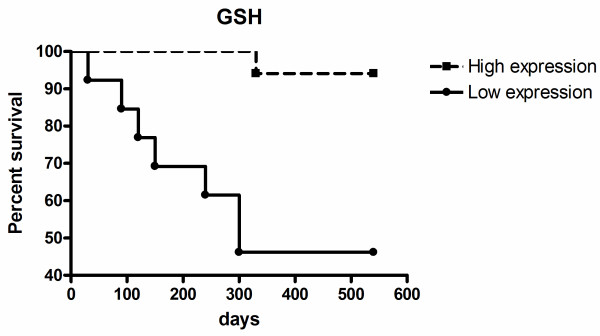
**Survival rates for female dogs according to GSH expression.** Overall survival for high GSH expression (dotted line) versus low GSH expression (continuous line) (selected cut-off = M.O.D. = 197 a.u.) (p = 0.002 / OR. 0.07 / C.I. 95%: 0.02 to 0.43). OR = odds ratio; C.I. = confidence interval.

We found that the overall survival of patients with low GSH expression was significantly shorter than patients with high GSH expression (*P* = 0.002; Figure 2). The median survival time of dogs that died was 6.5 months. There was no correlation between GSTpi and GSH-Px expression and overall survival (*P* > 0.05; data not shown).

### In vitro study

**Figure 3:**
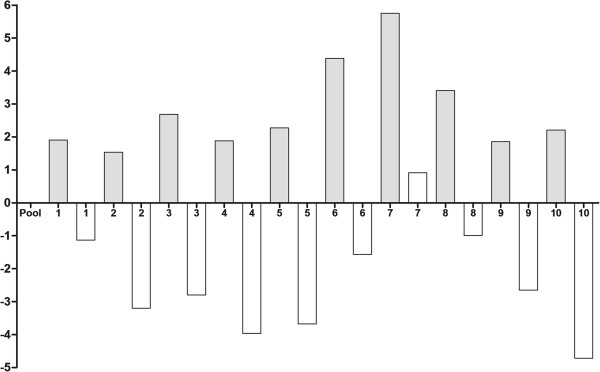
**Quantitative gene expression of GCLC, a synthesizer of GSH, in mammary tumor cells of female dogs after treatment with doxorubicin.** The treated group (white bars) showed underexpression of GCLC gene compared with the control group [untreated samples (gray bars)] (p = 0.0001). Value of gene expression in log3.

**Figure 4:**
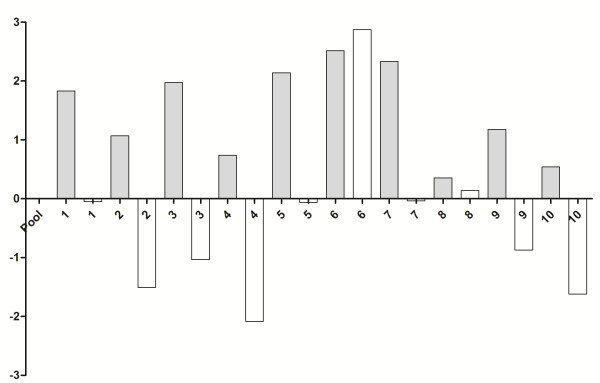
**Quantitative gene expression of GSS, a synthesizer of GSH, in mammary tumor cells of female dogs after treatment with doxorubicin.** The treated group (white bars) showed underexpression of GSS gene compared with the control group [untreated samples (gray bars)] (p = 0.001). Value of gene expression in log3.

**Figure 5:**
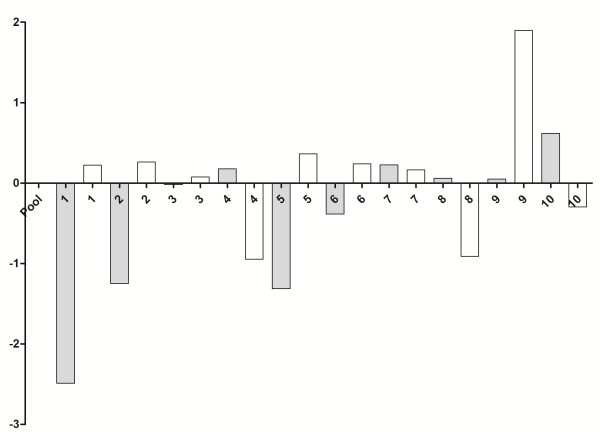
**Quantitative gene expression of GSH-Px in mammary tumor cells of female dogs after treatment with doxorubicin.** The treated group (white bars) compared with the control group [untreated samples (gray bars)] showed no significant difference in the GSH-Px expression. Value of gene expression in log3.

10 tumor specimens were used from the 30 samples used for immunohistochemistry, which were histopathologically identified as seven carcinomas in mixed tumors, two tubulopapillary carcinomas and one papillary carcinoma. After treatment with doxorubicin, all the samples showed underexpression of the GSH gene compared with the control group (without treatment) (*P* = 0.0001; Figure 3) and the GSS gene was also expressed more weakly in the presence of the drug in 90% of the samples (*P* = 0.001) (Figure 4). There was no significant statistical difference between GSH-Px gene expression in the control group relative to the group treated with doxorubicin (*P* > 0.05; Figure 5).

## Discussion

In this study, there was an increase in GSH expression in tumors without ulceration, not metastatic tumors and low mortality. Our results are in agreement with Buser et al. [12] who demonstrated that high levels of GSH, GSTpi and GSH-Px were associated with favorable clinical characteristics and good prognosis, whereas low levels of GSH and GST activity were associated with more aggressive or more advanced disease.

After 18 months, no significant results were observed when comparing the GSTpi and GSH-Px expression with metastasis and death in our dogs. Our results are in agreement with Jardim et al. [1] where no statistical correlation was found between GSTpi expression and local recurrence and/or metastasis either.

In this study, we verified the influence of chemotherapy with doxorubicin on the GSH and GSH-Px genes expression. According to cell culture growing curves, doxorubicin efficiently causes tumor cell death.

Moreover, doxorubicin treatment reduces the expression of the genes GCLC and GSS, respectively, in treated tumor cells over untreated tumor cells. GCLC sensitizes the enzyme glutamate cysteine ligase to catalyze the first step for GSH synthesis and GSS codes for glutathione synthetase catalyzing the second and last step of synthesis of GSH. Together, these data support the notion that doxorubicin reduces a pattern of gene expression associated with modulation of the antioxidant capacity of mammary tumor cells.

Many studies have shown alterations in GSH activity after *in vitro* treatment with doxorubicin or similar drugs. Our results agree with Ozkan and Fiskin [13], in which epirubicin - a structural analog of doxorubicin - decreases GSH activity after 24 h in cultured mammary neoplastic cells.

Some studies suggest that reactive oxygen species (ROS) production in doxorubicin treatment is responsible for cytotoxicity in neoplastic cells, which could induce the synthesis of antioxidant enzymes, such as GSH and GSH-Px, making the neoplastic cells resistant to oxidative damage [14]. In the same way, Han et al. [15] found greater sensitivity to doxorubicin when the GSH levels decreased in the mammary carcinoma cell line (MCF-7). However, in contrast, Di et al. [16] demonstrated that GSH overexpression did not prevent tumor cell apoptosis after treatment with doxorubicin, suggesting that the cytotoxicity of the drug might not be wholly related to ROS production.

Additionally, the involvement of GSH in the therapeutic response to doxorubicin is controversial. Some authors suggest that GSH inhibition leads to the drug accumulating in the nucleus of the tumor cells [17,18], whereas others suggest that resistance to doxorubicin and other similar drugs is not directly related to the binding of the drug to GSH [14].

Exactly what intracellular alterations are responsible for the MDR phenotype in neoplastic cells remain largely unknown. There is a suggestion that high concentrations of GSH, GSH-Px and GSTpi, independent of other intracellular alterations, do not decisively contribute to MDR [19,20].

The resistance to the antineoplastic agent cisplatin, for instance, has been associated to the high expression of the enzyme gama glutamyl transpeptidase (GGT) localized at the cell membrane, which is responsible for its degradation. Thereby, despite the fact that cytotoxic treatment decreases intracellular GSH concentration, this enzyme hydrolyzes serum GSH, releasing the necessary amino acids for intracellular GSH replenishment [21]. The enzyme GGT is mainly found in liver and kidney cells; however, resistance to drugs due to high levels of this enzyme has been found in many cancer types including some mammary tumors [21].

In this context, there are many studies trying to identify agents that can increase the efficiency of conventional drugs and/or reverse the MDR phenotype in neoplastic cells [22,23]. Despite the correlation between high expression of these genes and the MDR, some studies suggest that using agents that increase GSH, GSH-Px and GSTs levels during drug treatment is a promising way to reduce oxidative stress, thus reducing the collateral effects of chemotherapy in breast cancer patients [24-26].

The mammary tumors cultivated in vitro showed underexpression of the GSH coding genes and no alteration to the expression of GSH-Px, after treatment with doxorubicin. This can be explained by the interaction between these two enzymes in the antioxidant defense of tumor cells. GSH-Px has selenium at its catalytic site and uses GSH as an electron donor for the reduction of H_2_O_2_ to H_2_O [12,27]. So the results show that GSH and GSH-Px don’t provide adequate defense against oxidants, rendering them sensitive to drugs.

In this context, it is necessary to clarify the mechanism of these enzymes in cell resistance and their true prognostic value [28,29]. Knowledge of GSH expression and its enzymes in a wide range of biology processes is expanding. Advances in analytical techniques and protein localization will present many opportunities for the development of therapeutic interventions in cancer and other diseases related to levels of oxidative stress [5].

## Conclusion

High expression of GSH in neoplastic mammary cells from female dogs is associated with the best clinical outcome, including a greater survival time. Therefore this high expression plays an important role in the clinical outcome of patients with mammary tumors and can be used as prognostic marker for mammary neoplasms in female dogs.

Treatment of mammary neoplastic cells with doxorubicin *in vitro* is an efficient way to induce cell death, reducing the expression of the *GCLC* and *GSS* genes that are related to antioxidant production. Thus they can be considered potential predictive markers on the therapeutic feedback to breast cancer. More studies, using large cohorts in future studies, are needed to confirm the prognostic value of these markers in the mammary tumor progression in female dogs.

## Methods

### Immunohistochemistry study

#### Sample characterization

Tumor fragments were collected from 30 female dogs with mammary neoplasia that were brought to the veterinary clinics (São José do Rio Preto and region) during the years of 2009 and 2010. After tumor excision, the animals were followed-up from 1–18 months, with a median of 540 days. During follow-up time, the vets evaluated tumor metastasis and recurrence, as well as the cause of death of the animal.

For histopathologic diagnostics, the tumor biopsies collected were classified according to Misdorp et al. [30] by the AFIP (Armed Forces Institute of Pathology). The parameters employed for the classification of clinical tumor staging were in accordance with the TNM system (size, lymph node involvement, metastasis) established by WHO for canine mammary gland tumors modified [31], which recommended tumor mass size (T) – T1: < 3 cm - T2: between 3 and 5 cm - T3: > 5 cm; lymph node involvement (N) - N0: no apparent involvement - N1: unilateral involvement - N2: bilateral involvement; and distant metastasis (M) - M0: no evident metastasis - M1: distant metastasis including non-regional lymph nodes. Clinical staging was assigned as I, II, III or IV according to the tumor extension and established prognosis.

The presence of local tumor recurrence, metastasis and death were described and the overall survival was determined from the date of diagnosis until last follow-up or death. The cause of death was evaluated by the attending veterinarian and only female dogs that died of the illness were included in the group for the study. The dogs that had died of respiratory failure were diagnosed with lung metastasis as shown by X-ray. This study was approved by the Ethical Committee of the Faculdade de Medicina de São José do Rio Preto (Protocol number 3945/2009).

#### Immunohistochemistry technique

For immunochemistry, tumor samples were embedded into paraffin blocks and cut to give 3 μm sections. The samples were prepared on silanized glass slides before the paraffin was removed, the sections rehydrated in an ascending range of alcohol concentrations and incubated with 3% hydrogen peroxide for 30 min to block endogenous peroxidase activities. Antigenic recovery was made in a recipient at 95°C in buffer for 35 min for each specific antibody. The GSH primary antibody was diluted in the proportion of 1:100, GSTpi 1:4000 and GSH-Px 1:1200 in bovine serum albumin solution (BSA). After cooling, the slides were covered with BSA for 30 min and incubated at 4°C overnight with the antibodies.

After they had been washed with phosphate buffer (PBS) for 15 min and incubated with Easy Path kit (Erviegas, São Paulo, Brazil), composed of the secondary antibody “anti-mouse, rabbit and goat immunoglobulin with Biotin” for one h and “streptoavidin complex with peroxidase” for 30 min, followed by washes with PBS for 15 min, 0.5% of 3,3′-diaminobenzidin-tetra-hydrochloride (DAB, Signet® Laboratories, Dedham, MA, USA) was applied to the slides for 2–5 min at 20-22°C. The slides were counterstained with Harris hematoxilin for 40 min. Negative controls were obtained by the omission of the primary antibody, and one sample of prostate was used as an internal control in each assay.

#### Immunohistochemistry quantification

The slides were photographed and the proteins quantified by the AxioVision software at 40× magnification under an AXIOSKOP2 Zeiss microscope. For each sample, three regions of the tumor tissue were selected and 20 points of the tumor cells were marked in each region. In this way, 60 different points were analyzed in each sample to obtain an average relative intensity of immunoreactivity. The values were given in arbitrary units (a.u.) and the mean optical density (M.O.D.) showing the specific immunostaining intensity at immunoreactive areas.

### In vitro study

#### Sample characterization

The *in vitro* study was performed with tumor biopsies of 10 dogs out of the 30 with mammary tumors from the immunohistochemistry study. The tumor biopsies were sliced into microfragments and incubated at 37°C in 5% CO2 in RPMI1640 (Cultilab) supplemented with 20% BSA, 1% penicillin/streptomycin and 1% L-glutamine. The cells were cultured until there was 80% confluence and then immunocytochemistry was carried out with the primary antibodies anti-cytokeratin, anti-vimentin and anti-calponin for epithelial origin confirmation.

#### In vitro cell treatment

The cells from each tumor biopsy were divided into two groups: control (untreated) and that treated with 0.2 mg of the drug (doxorubicin, Adriamycin) for 24 h. At the end of the treatment, cell viability was verified by cell counting with the Neubauer chamber after staining with trypan blue dye. The cells were submitted to immunocytochemistry for confirmation of the epithelium origin, with anti- cytokeratin antibody, resulting in a positive protein staining.

#### Quantitative RT-PCR (qPCR)

Total RNA was extracted from the cell cultures with TRIZOL reagent (Invitrogen). Each sample of total RNA was subjected to reverse transcription using a High Capacity cDNA kit (Applied Biosystems).

The quantitative PCR (qPCR) was performed according to Bustin et al. [32].

The polymerase chain reaction (qPCR) was made in triplicate using the equipment Step One Plus (Applied Biosystems). The reaction had a final volume of 20 μL, with 10 μL of Power SYBR Green PCR Master Mix (Applied Biosystems), 250 μL of each primer, and 10 ng of cDNA. The qPCR conditions were 50°C for 2 min, 95°C for 10 min, followed by 35 cycles of 95°C for 15 s and 60°C for 1 min. The dissociation curve was analyzed to confirm the desired genetic product: one cycle of 95°C for 15 s, 60°C for 1 min, and 95°C for 15 s.

Endogenous control of the genes *RPS19* and *RPL8* were used for normalization. The transcription level was calculated by the 2-ΔCt method [33]. The Ct number (relative abundance) was calculated using the 2-ΔΔCT formula and was plotted as mean ± SD of the triplicates reaction.

The genes were selected from PUBMED. The *GCLC* and *GSS* genes were selected because they synthesize GSH, and the gene *GST-Pi* was not used in this study because its sequence was not available for the canine specie. Primers were designed with PRIMER3 software. The primers used for amplification were: *GCLC* sense (5′-CCAAGTCCCTCTTCTTTCCTG-3′) and antisense (5′-CGGAGACGGTGT ATTCTTGTC-3′) [GenBank: NG_012071.1]; *GSS* sense (5′-AGCCAATGCTCTGGTGCTAC-3′) and antisense (5′-ACCTTCGACGGATTACATGG-3′) [GenBank: NG_008848.1]; *GSH-Px* sense (5′-GGCATCAGGAAAACGCTA AG-3′) and antisense (5′-CCTCGCACTTCTCAAAAAGC-3′) [GenBank: NG_012264.1]; *RPS19* (internal control), sense (5′-CCTTCCTCAAAAAGTCTGGG-3′) and antisense (5′-GTTCTCATCGTAGGGAGCAAG-3′) [GenBank: NG_007080.2]; and *RPL8* (internal control), sense (5′- CCATGAATCCTGTGGAGC-3′) and antisense (5′-GTAGAGGGTTTGCCGATG-3′) [GenBank: NC_000008.10]. They were classified as subexpressed [samples with values lower than -1 (Log3)] and superexpressed [samples that presented values higher than -1 (Log3)].

### Statistical analyses

All the analyses were done with the assistance the GraphPad Prism4 and StatsDirect software. The female dogs were grouped according to their clinical-pathological variables: age, time course, histological type, clinical staging, number of nodules, tumor ulceration, tumor vascularization, metastasis and death. The average referred to the densitometry, and quantification for the different mammary tumor groups was compared with Student’s *t* test or ANOVA, followed by Bonferroni test. The values were expressed as mean ± S.E.M.

The cutoff for the death risk was determined by the ROC curve. This analysis graphically represents the comparison between the sensitivity distribution specifically for each factor, or the true positive index or the false positive index. Whenever the ROC curve is closer to the left superior quadrant, the test variable is more precise, since the positive value (sensitivity) would be closer to 1 and false positive (specificity) closer to 0 [34]. Survival curves were plotted using the Kaplan-Meier method and the differences between the curves were evaluated by a log-rank test and hazard function. All the 30 female dogs of the study were included in this analysis, and each death case is represented by the decrease of the survival percent in the graph in the correspondent day of the death. The overall survival was determined from the date of diagnosis until last follow-up or death.

To determine the possible association between the relative differences of the genetic expression of the groups, the Man-Whitney *U* test was used. *P* < 0.05 was adopted as significant.

## Abbreviations

°C: Degrees celsius; BP: Base pare; BSA: Bovine serum albumin; cDNA: Complementary DNA; Ct: Cicle Threshold; DAB: Diaminobenzidine; EDTA: Ethylenediamine tetraacetic acid; GCLC: Glutamate-cysteine ligase; GSH: Glutathione; GSS: Glutathione synthetase; GST: Glutathione S Tranferase; GSTpi: Glutathione S Tranferase PI; GSH-Px: Glutathione peroxidase; HE: Hematolxilin-eosin; C.I.: Confidence interval; kDa: Kilodaltons; MDR: Multiple drug resistence; MG: Miligram; M.O.D.: Mean optical density; OR: Odds ratio; PBS: Phosphate buffer solution; PCR: Polimerase chain reaction; qPCR: Quantitative real time PCR; ROC: Receiver operating characteristcs; ROS: Reactive oxygen species; RPL8: Ribosomal protein L8; RPS19: Ribosomal protein S19; S.E.M.: Standard deviation; SH: Sulfhydryl group; TNM: Clinical staging system: T = tumor, N = node, M = metastasis; a.u.: Arbitrary units

## Competing interests

The authors declare that they have no competing interests.

## Authors’ contributions

CL and DAPCZ conceived the study and drafted the manuscript. CL e GBG conducted the collect of mammalian tumor from bitches and the histopathological procedure. CL e BVJ performed the cellular culture and the chemotherapy treatment. MGM performed the statistical tests of the immunohistochemistry. VRR did the immunohistochemistry procedure. CL e JGO performed the qPCR and the statistical analyses. All authors have read and approved the final manuscript.
